# Reply to Tjalma, W.A. Comment on “Gentile et al. Superior Survival and Lower Recurrence Outcomes with Breast-Conserving Surgery Compared to Mastectomy Following Neoadjuvant Therapy in 607 Breast Cancer Patients. *Cancers* 2025, *17*, 766”

**DOI:** 10.3390/cancers17122009

**Published:** 2025-06-17

**Authors:** Damiano Gentile, Jacopo Canzian, Erika Barbieri, Simone Di Maria Grimaldi, Rita De Sanctis, Corrado Tinterri

**Affiliations:** 1 Breast Unit, IRCCS Humanitas Research Hospital, Via Manzoni 56, Rozzano, 20089 Milan, Italy; erika.barbieri@cancercenter.humanitas.it (E.B.); simone.dimariagrimaldi@cancercenter.humanitas.it (S.D.M.G.); corrado.tinterri@hunimed.eu (C.T.); 2 Department of Biomedical Sciences, Humanitas University, Via Rita Levi Montalcini 4, Pieve Emanuele, 20090 Milan, Italy; jacopo.canzian@cancercenter.humanitas.it (J.C.); rita.de_sanctis@hunimed.eu (R.D.S.); 3 Medical Oncology and Hematology Unit, IRCCS Humanitas Research Hospital, Via Manzoni 56, Rozzano, 20089 Milan, Italy

We would like to thank Dr. Wiebren A Tjalma for his interest in our work and his thoughtful comments on our recently published article evaluating long-term outcomes in patients with breast cancer (BC) undergoing breast-conserving surgery (BCS) versus mastectomy after neoadjuvant therapy (NAT) [1,2]. We appreciate the opportunity to clarify some of the points raised and to expand upon our methodology and clinical rationale.

Dr. Tjalma raises concerns regarding the proportion of patients who did not undergo preoperative mammography, citing a figure of 25%. We would like to respectfully correct this statement. As indicated in Table 1 of our manuscript, 457 of the 607 patients (75.3%) underwent mammography as part of their preoperative assessment. It is important to consider, however, that 42 patients in our cohort were younger than forty years old, an age group for which mammography is not routinely recommended for local radiologic staging due to the reduced sensitivity of the technique in dense breast tissue. Thus, the proportion of patients not undergoing mammography due to clinical appropriateness is reduced to 17.8%, rather than 25%. We believe this clarification is essential in properly contextualizing adherence to radiologic staging protocols.

Regarding magnetic resonance imaging (MRI), Dr. Tjalma notes that the percentage of patients undergoing preoperative MRI was unspecified. However, Table 1 reports that 57.2% of patients underwent MRI before surgery. While we acknowledge that this rate is below the current EUSOMA target, we emphasize that our dataset spans 17 years, from 2006 to 2023. Before 2016, MRI was not yet widely adopted for routine staging after NAT, particularly in several Italian regions. As our center is a high-volume referral Breast Unit, our patients come from across the country, and MRI availability was not always guaranteed during earlier years. Therefore, the observed rate should be interpreted in the context of temporal trends in imaging availability and clinical practice evolution.

Dr. Tjalma also comments that “the approach to axillary management is notably absent.” With respect, we would like to highlight that axillary surgery was reported in detail in Table 1 under “Surgical treatment.” Specifically, sentinel lymph node biopsy (SLNB) not followed by axillary lymph node dissection (ALND) was performed in 345 patients (56.8%), SLNB followed by ALND in 87 patients (14.3%), and direct ALND in 175 patients (28.9%). These data provide a comprehensive overview of axillary management in our cohort. Moreover, we do not routinely adopt targeted axillary dissection at our Breast Unit [3], which remains a matter of institutional preference and guideline interpretation.

With respect to HER2-targeted therapy, Dr. Tjalma notes a numerical discrepancy between HER2-positive cases (n = 266) and trastuzumab administration (n = 267). Upon re-evaluation, we confirm that this was not an error in data reporting. One patient initially diagnosed as HER2 2+ with FISH amplification on core needle biopsy received trastuzumab as part of neoadjuvant treatment. However, the final surgical pathology revealed a lack of amplification upon retesting. This kind of discordance between biopsy and surgical specimens, although infrequent, is well-documented and was managed accordingly by discontinuing anti-HER2 therapy postoperatively.

Dr. Tjalma further suggests that HER2-positive and triple-negative tumors would be expected to be overrepresented. While this is generally valid, our cohort includes a substantial proportion of luminal-like patients with BC, the vast majority of whom presented with clinically positive nodal disease and were referred to NAT as part of a downstaging strategy. This contributes to the observed pathologic complete response (pCR) rate of 25.2%.

As for the use of metallic clips rather than hydromarkers for tumor localization, this represents an institutional choice based on available resources. While hydromarkers offer advantages in post-NAT MRI assessment, metallic clips remain widely utilized and effective. The presence of susceptibility artifacts on MRI did not significantly affect treatment planning in our experience.

Finally, we wish to address the broader implication raised by Dr. Tjalma, namely, whether BCS is feasible and safe following NAT. As demonstrated in our study, BCS was not only feasible in 54.7% of patients but was also associated with superior oncologic outcomes compared to mastectomy across all survival metrics. If these outcomes were achieved despite the limitations of radiologic staging noted by Dr. Tjalma, one could indeed speculate that improved access to advanced imaging would only further enhance the safety and efficacy of BCS!

In conclusion, we reiterate our position that BCS, when feasible, represents not only an oncologically safe but potentially superior option following NAT. We strongly support its adoption through individualized surgical planning, multidisciplinary evaluation, and continuous adherence to evolving evidence and guideline recommendations.
